# Physicochemical, Rheological, and Sensory Characteristics of Yogurt Fermented by Lactic Acid Bacteria with Probiotic Potential and Bioprotective Properties

**DOI:** 10.3390/foods12132552

**Published:** 2023-06-29

**Authors:** Ramize Hoxha, Yana Evstatieva, Dilyana Nikolova

**Affiliations:** Department of Biotechnology, Faculty of Biology, Sofia University “St. Kliment Ohridski”, 1164 Sofia, Bulgaria; rhodzha@uni-sofia.bg (R.H.); y.evstatieva@biofac.uni-sofia.bg (Y.E.)

**Keywords:** yogurt, fermentation, probiotic strains, *Lactobacillus delbrueckii* subsp. *bulgaricus*, *Lactiplantibacillus plantarum*

## Abstract

The applicability of two lactic acid bacterial strains with probiotic potential and bioprotective properties as additions in the starter culture in yogurt fermentation was examined. The studied strains, *Lactobacillus delbrueckii* subsp. *bulgaricus* KZM 2-11-3 and *Lactiplantibacillus plantarum* KC 5-12, inhibited the growth of *Kluyveromyces lactis*, *Kluyveromyces marxianus*, and *Saccharomyces cerevisiae*. The strain *L. delbrueckii* subsp. *bulgaricus* KZM 2-11-3 directly inhibited *Escherichia coli*. The important characteristics for the quality of the yogurt product, such as physicochemical parameters during fermentation and storage, rheological characteristics, and sensory changes during the storage of samples were determined. The yogurt samples with the strains did not differ in most parameters from the control yogurt with the commercial starter. The added strains showed stable viability in the yogurt samples during storage. The yogurt sample with *L. delbrueckii* subsp. *bulgaricus* KZM 2-11-3 and the sample with both strains based on the total evaluation were very similar to the control yogurt with the commercial starter. Using these strains as probiotic supplements to enrich the starter cultures in yogurt production will contribute to developing new products with benefits to human health.

## 1. Introduction

Functional food has many definitions, but according to the European consensus, functional food, besides its basic nutritional value, improves health benefits and reduces the risk of diseases (European Commission) [1]. A functional food can be a natural food or one to which components have been added, removed, or modified [2]. Traditional dairy foods can be considered functional foods as they have nutritional value and more importantly, they impart health benefits [3]. Among the functional milk-based products is yogurt. Yogurt is one of the most popular products originating from countries around the Balkans and Eastern Mediterranean Sea and is widely consumed worldwide. It was produced based on the knowledge and processes that were inherited from the ancestors. Yogurt is a result of the fermentation process of living microorganisms, specifically lactic acid bacteria (LAB), which ferment lactose into lactic acid, causing the coagulation of milk. LAB present in milk during fermentation play a very important role in enriching it with nutritional values. Microorganisms enable the enrichment of milk with lactic acid, peptides, and amino acids with antimicrobial [4] and antioxidant activities [5], as well as break down the milk fat into free fatty acids [6] and produce compounds in the yogurt matrix that contribute to the aroma and taste of yogurt [7,8]. The combination of *Streptococcus thermophilus* and *Lactobacillus delbrueckii* subsp. *bulgaricus* strains is the most common starter culture to produce yogurt [4,9]. The first researcher to discover *L. bulgaricus* was a Bulgarian scientist, Stamen Grigorov, in 1905. The lactic acid bacterium was named *Bacillus bulgaricus*. In 1909, Illya Metchnikoff, a Nobel Prize-winning scientist, suggested that the longevity of the Bulgarian population was associated with the lactobacilli present in yogurt [10]. *L. bulgaricus* has attributes enabling its use as a probiotic, showing health benefits in the hosts [11]. In addition to these two species, other species are also used as starter cultures [12]. *Lactiplantibacillus plantarum* can be used as a starter culture, enriching the yogurt with amino acids, volatile flavor compounds, and unsaturated fatty acids [13,14]. Numerous studies have shown that yogurt has different beneficial effects on human health, such as antimicrobial and antioxidant effects [15,16,17], helps against gastrointestinal diseases [18], is anti-inflammatory [19], acts against high blood pressure [17,20] helps the immune system [21], reduces the risk of osteoporosis [22], and helps with cardiovascular diseases and diabetes [22,23].

The steps of the production process such as the thermal treatment of milk, incubation conditions during fermentation, and the cooling process [24], as well as the composition of the milk and the types of bacterial cultures, are the main aspects of the texture of yogurt [25]. Knowledge of rheological properties is of particular importance in product quality control. There are several factors that affect viscosity. The type of strain used mainly acts as an important factor in the viscosity of the yogurt. The fermentation rate (low incubation temperature) results in lower viscosity. The decrease in syneresis brings an increase in viscosity [24]. The acidification caused by LAB producing lactic acid is an important mechanism for the process of yogurt production, which leads to a decrease in pH. While the pH of milk decreases, milk proteins aggregate to form yogurt gel [26]. Redox potential (RP) is attracting attention as an important component in fermentation processes. According to Martin et al., RP has been proven to stimulate the production of aroma compounds [27]. Yogurt is composed of water, proteins, polysaccharides, and fat and is a rich source of calcium, magnesium, vitamin B, etc. [22,28]. The protein–water interaction (protein solubility) is considered to be one of the most important functional attributes of proteins because it affects the structure, color, emulsification, foaming, and sensory properties of food products. High solubility is desirable to achieve the required degree of gelation. Protein solubility depends on the amino acid composition, molecular weight, surface characteristics of the constituent amino acids, and environmental factors such as pH, temperature, and ionic strength [29]. Zayas, (1997) defined the water-holding capacity (WHC) of foods as the ability to hold their own and added water during the application of forces, i.e., pressing, centrifugation, or heating; the WHC plays a major role in the formation of the food texture. The WHC of a protein gel is a vital consideration in yogurt production because it is related to viscosity and syneresis, which is due to the internal instability of the gel (water released or expelled from the three-dimensional structure of proteins), resulting in water loss after some storage time [30]. Syneresis is the whey separation ability and is undesirable for yogurt. Syneresis is considered a texture defect in yogurt. The restructuring of the matrix of casein micelles leads to the removal of water from the structure. This is the main cause of syneresis. Factors that have a significant impact on the texture and removal of whey in yogurt are the composition of the base milk, the fermentation process, the strains used, the type of acidification kinetics, and the post-fermentation treatment [31]. All of these parameters are related to each other, and together they contribute to the formation of the final product, with the corresponding physicochemical, rheological, and sensory characteristics.

As mentioned above, one of the factors that affects the product production process is the bacterial culture [25]. The presence of different strains in a product influence the parameters mentioned above, which means that the product has different characteristics depending on the strains used [13,14]. Increasing attention to probiotic yogurt as a functional food with beneficial effects for humans directs scientific researchers to research product characteristics when probiotics are present in the product. For the development of new dairy products with applied probiotic auxiliary cultures, the most important factors that must be taken into account are the effect of probiotics in the fermentation process, the quality of the product, and the final acceptability for consumers [14]. 

In the current study, the strain *L. delbrueckii* subsp. *bulgaricus* KZM 2-11-3, an isolate from traditionally prepared yogurt, and *Lpb. plantarum* KC 5-12, an isolate from artisanal cheese, were preselected as strains with probiotic potential and bioprotective properties [32]. In both strains, a well-defined effect of the viral replication inhibition of human alpha herpesvirus HHV-1 and HHV-2 with a high selective index was observed [33]. Both strains have shown antibacterial activity against a wide range of gram-positive and gram-negative pathogenic bacteria and antifungal activity against filamentous molds associated with food contamination. The strain *Lpb. plantarum* KC 5-12 has a very good ability to survive under different conditions of the gastrointestinal tract (GIT) such as pH 2 and the presence of enzymes and bile salts, while the strain *L. delbrueckii* subsp. *bulgaricus* KZM 2-11-3 has a good ability to exhibit auto-aggregation and hydrophobicity and has adhesive potential, such as binding to mucin, characteristics that determine their role in competition with pathogens and their probiotic potential [32]. 

The aim of this work is related to studying the applicability of the two selected strains in yogurt starter cultures to enrich the starter culture with strains that have health benefits for consumers. The important characteristics for the quality of the yogurt product, such as physicochemical parameters during fermentation and storage, rheological characteristics, sensory analysis, and strain viability during the storage of samples, were determined. 

## 2. Materials and Methods

### 2.1. Co-Cultivation of Escherichia coli with LAB Strains in Milk

The co-cultivation of test bacterium *Escherichia coli* ATCC 25922 with two selected LAB strains, *L. delbrueckii* subsp. *bulgaricus* KZM 2-11-3 and *Lpb. plantarum* KC 5-12, was determined according to Fijan et al. [34] with modifications. In brief, 19 mL of sterilized milk (with 3% fat) was initially inoculated with 2.5% of lactic acid bacteria (initial concentration 10^8^ CFU/mL). Then, 1 mL of *E. coli* overnight culture standardized to 0.8 Mac Farland (10^8^ CFU/mL) was inoculated. *E. coli* samples inoculated in milk without the presence of LAB and with the presence of a 1% commercial starter culture (LB Bulgaricum, Sofia, Bulgaria) were used as control variants. The fermentation was carried out overnight at 37 °C in an incubator (Binder, Tuttlingen, Germany). The enumeration of test bacteria was carried out immediately after inoculation, after the fermentation, and after 5 days of storage at 4 °C. The colony counting after serial dilutions was performed in Petri dishes containing HiCrome *E. coli* Agar (HiMedia, Mumbai, India) selective medium for *E. coli* following incubation at 37 °C for 24 h. 

### 2.2. Antifungal Activity against Yeasts

Antifungal activity was determined according to Fayyaz et al. [35] with modifications. Cell-free supernatants (CFSs) from 24 h cultures of the two isolated strains were used against yeasts *Kluyveromyces lactis* 1470, *Kluyveromyces marxianus* var t3, and *Saccharomyces cerevisiae* NBIMCC 537. Selected strains cultivated for 24 h in de Man, Rogosa, and Sharpe (MRS) broth (Himedia, Mumbai, India) were centrifuged (6000× *g*, 10 min, 4 °C), and the supernatants were filtered through 0.20 µm membrane filters. Then, 950 µL of the isolated supernatants and 50 µL of the yeast suspension (10^6^ CFU/mL) were dispensed in 24-well plates (Costar, Corning Incorporated, MA, USA). The plates were placed in a shaker (Lauda-GFL 3033, LTF Labortechnik GmbH & Co. KG, Wasserburg, Germany) at 180 rpm/min at 30 °C for 72 h. The growth of the yeasts was measured using a SPECTROstar^®^ Nano Microplate Reader (BMG LABTECH, Ortenberg, Germany) at 600 nm. Inoculated yeasts in malt extract broth were used as positive controls (Oxoid, Basingstoke, UK). The yeast growth was calculated according to [36] as OD_t_ − OD_0_, where the OD_t_ is the absorbance after a certain incubation time, and OD_0_ is the absorbance at the start. The yeast growth in the control was considered to be 100% growth. Based on this, the percentage yeast growth inhibition was calculated.

### 2.3. Yogurt Preparation

The yogurt samples were prepared according to Fayyaz et al. [35] with modifications, using pasteurized cow’s milk with 3% fat content. The commercial starter culture of yogurt (LB Bulgaricum, Sofia, Bulgaria) and the selected strains (*L. delbrueckii* subsp. *bulgaricus* KZM 2-11-3, *Lpb. plantarum* KC 5-12) at 10^8^ CFU/mL were inoculated in tempered milk at the concentrations in Table 1. The sample with inoculation of 1% commercial starter culture (LB Bulgaricum, Sofia, Bulgaria) was used as a control. The fermentation process lasted 5 h at 41 °C in an incubator (Binder, Tuttlingen, Germany), and then the samples were stored at 4 °C for 28 days.

### 2.4. Determination of pH, RP, and Titratable Acidity (TA)

The pH and RP (mV), were measured with a digital pH meter (FiveEasy F20, Mettler Toledo^®^, Greifensee, Switzerland) during the fermentation process every hour and during the storage of samples on days 0, 7, 14, 21, and 28. For the measurement of TA (°T), 10 mL of samples was mixed with 10 mL of distilled water using as an indicator 0.5% phenolphthalein and titrated with 0.1 N NaOH [37]. The measurements were made with a digital burette (ISOLAB Laborgeräte GmbH, Eschau, Germany) at the same experimental points as pH and RP during the process of fermentation and the storage time.

### 2.5. Determination of the Water-Holding Capacity and Syneresis

The WHC was determined via centrifugation at 4000× *g* for 20 min at 10 °C following the method according to Fayyaz et al. [35]. The initial yogurt weight and supernatant weight were measured. The WHC was calculated following Parvarei et al. [38]: WHC (%) = [(Yogurt weight − Supernatant weight)/Yogurt weight] × 100.

Syneresis was determined according to [38] with modification, where 10 g of yogurt samples was centrifuged at 260× *g* for 10 min at 4 °C. The weight of separated whey was measured and the percentage of syneresis determined following the formula: Syneresis (%) = (whey separated/10) × 100.

### 2.6. Apparent Viscosity

Apparent viscosity was determined according to Yan et al. [39] with modification. The apparent viscosity was measured with an NDJ-5S digital viscometer (Shanghai Drawell Scientific Instrument Co., Ltd., Shanghai, China) using spindle No. 4, and the shear velocity was 30 rpm for 60 s. The apparent viscosity was expressed in cP (centipoise). 

### 2.7. Enumeration of Bacteria in Yogurt

One gram of each yogurt sample was diluted with 10 mL PBS, and serial dilutions were made according to [40]. Then, 1 mL of the corresponding dilution was spread on MRS agar for the enumeration of LAB strains after 48 h of incubation at 41 °C. This method was repeated on days 0, 7, 14, 21, and 28, and the number of bacteria was calculated as CFU/g. 

### 2.8. Sensory Analysis

Sensory analysis of the yogurt was performed by a panel (*n* = 15) pre-acquainted with the sensory characteristics of yogurt according to the National Standard for Bulgarian Yogurt (BDS 12:2010) [41]. The evaluation of yogurt samples was performed for the determination of the compliance of seven sensory indicators according to BDS 12:2010. Sensory analysis examines the indicators (surface, color, presence of liquid above the surface, structure, homogeneity, aroma, and taste) on a scale from 0 to 5 for each indicator. When the indicators corresponded to the requirements of the standard, the maximum number of points was given, with fewer points given when there were deviations from the standard. The total number of points based on the seven indicators was 35, when each of them was evaluated with the maximum number of 5 points. The conditions for carrying out the sensory analysis of the yogurt were in accordance with the requirements of the Bulgarian National Standard for the sensory evaluation of milk products (BDS 15612:1983) [42]. All samples were coded and presented in sterile individual containers in an amount of 50 mL of yogurt. The sensory analysis of the yogurt samples was determined during storage (0, 7, 14, 21, and 28 days).

### 2.9. Statistical Analysis

All experiments were performed in triplicate. Results are presented as the mean ± standard deviation (SD). One-way analysis of variance (ANOVA) was applied using the Tukey test for comparison of the means of yogurt samples during the storage period (** *p* < 0.01 and * *p* < 0.05). A Pearson correlation was carried out to determine the relationship between the characteristics of the yogurt samples with the correlation coefficient (r) > 0.5.

## 3. Results and Discussion

### 3.1. Inhibition of Escherichia coli

*E. coli* is a bacterium that usually colonizes the intestines of warm-blooded organisms (humans and animals). Primary sources are raw or undercooked meat products, raw milk, and vegetable contamination. Some of the strains of *E. coli* can cause serious diseases [43]. LAB present in food products play a protective role because they are able to inhibit the growth of pathogenic bacteria [44,45]. 

**Figure 1:**
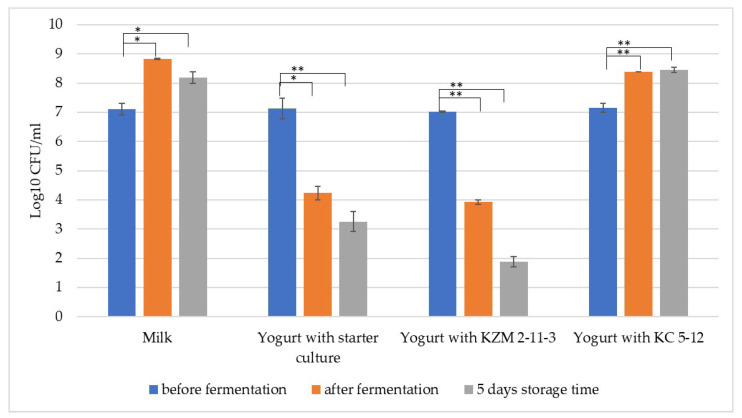
Growth inhibition of *Escherichia coli* ATCC 25922 by the selected strains. Error bars indicate the standard deviation of the means. One-way analysis of variance (ANOVA) was applied using the Tukey test for comparison of the means of variants of *E. coli* co-cultured with LAB in milk (** *p* < 0.01 and * *p* < 0.05).

Figure 1 presents the results of *E. coli* growth in yogurt inoculated with the lactobacilli strains and starter culture and in milk without the presence of LAB. The strain *L. delbrueckii* subsp. *bulgaricus* KZM 2-11-3 sensitively inhibited the growth of the test pathogen *E. coli*, with an almost 5-fold reduction in the product obtained after fermentation. After the storage of the product, the reduction in *E. coli* was more than 6-fold. In yogurt with strain *Lpb. plantarum* KC 5-12, no inhibitory effect was observed under these experimental conditions.

Different studies have shown that LAB can inhibit the growth of different strains of pathogenic and non-pathogenic *E. coli.* Oja et al. [44] reported that LAB inhibited diarrheagenic *E. coli* during co-culturing in yogurt. Fijan et al. [34], who co-cultured *E. coli* with probiotics, proved that single-strain probiotics had a greater effect in inhibiting the pathogen. By reducing the pH in yogurt, the antibacterial effect increases [45], and this may depend on the presence of the amount of lactic acid in the product and other organic acids. The viable cells of food-borne pathogens can be reduced by H_2_O_2_ accumulated by LAB [46]. Ortiz-Rivera et al. reported that the production of reuterin in a fermented milk product by *L. reuteri* inhibited pathogens and spoilage microorganisms, such as *E. coli* and other pathogens [47]. The studied strain *L. delbrueckii* subsp. *bulgaricus* KZM 2-11-3 can reduce the pH and has positive peroxidase activity [32], which could be the main reasons for its inhibitory activity against *E. coli* in yogurt.

For the yogurt variant with KZM 2-11-3 and the yogurt variant with the starter culture, there was a statistical difference in the viable cell counts of *E. coli* after fermentation and 5 days of storage compared with the viable cell counts of *E. coli* before fermentation, indicating a significant decrease in the number of cells (Figure 1). 

### 3.2. Antifungal Activity against Yeasts

In dairy products, yeasts such as *K. marxianus*, *K. lactis*, or *S. cerevisiae* are commonly present [48,49,50]. *Kluyveromyces* ssp. and *Saccharomyces* ssp. are frequent spoilers of fresh dairy products including fresh cheese and yoghurt [50,51,52]. Yeasts have the ability to metabolize milk components such as lactose, proteins, and fat. They use the lactose as a carbon source, competing with LAB for nutrients [51,53]. The yeasts contribute to the characteristics of the product in which they are present due to their ability to produce highly desirable aroma compounds, different from those of LAB, which lead to changes in the final product. LAB are particularly important in fermentation because, in addition to producing desirable acids and flavor compounds, they have the ability to inhibit the growth of undesirable organisms [54]. In our study, we aimed to prove that each of the studied strains could be incorporated into starter cultures for the production of yogurt that meets the requirements of the standard [41]. The presence of yeast imparts different sensory characteristics to the final products, and therefore, it was important to establish the effect of yeast growth inhibition.

Table 2 shows the results of the percentage growth inhibition of yeasts *K. marxianus* var t3, *K. lactis* 1470, and *S. cerevisiae* NBIMCC 537 in the CFSs of the two selected strains of LAB, *L. delbrueckii* subsp. *bulgaricus* KZM 2-11-3 and *Lpb. plantarum* KC 5-12. All three yeasts were inhibited by the CFSs of the LAB strains, in a specific and different way depending on the type of yeast and the LAB strain. The inhibition of yeast *K. lactis* occurred within 48 h. Meanwhile, for *K. marxianus* and *S. cerevisiae*, there was inhibition within 72 h by the CFSs of both strains. Both strains inhibited *K. marxianus* by more than 80% within 72 h and *K. lactis* by more than 60% within 48 h. Strain *Lpb. plantarum* KC 5-12 inhibited *S. cerevisiae* by about 70% and strain *L. delbrueckii* subsp. *bulgaricus* KZM 2-11-3 by about 30% within 72 h. The strain *Lpb. plantarum* KC 5-12, despite the fact that no inhibitory effect was observed for *E. coli*, was a good inhibitor of yeasts in foods, with an inhibitory effect greater than 60%. Other studies reported that *Lpb. plantarum* has antagonistic activity against yeasts *K. lactis*, *K. marxianus*, and *S. cerevisiae* as well as other yeasts [55,56]. As LAB have symbiotic relationships with yeasts present in different fermented products [57,58,59], the focus on antifungal activity may not be that high. *L. delbrueckii* has antifungal activity against pathogenic yeasts such as *Candida* ssp. [60].

### 3.3. Physicochemical Characteristics of Yogurt during Fermentation and Storage 

**Figure 2:**
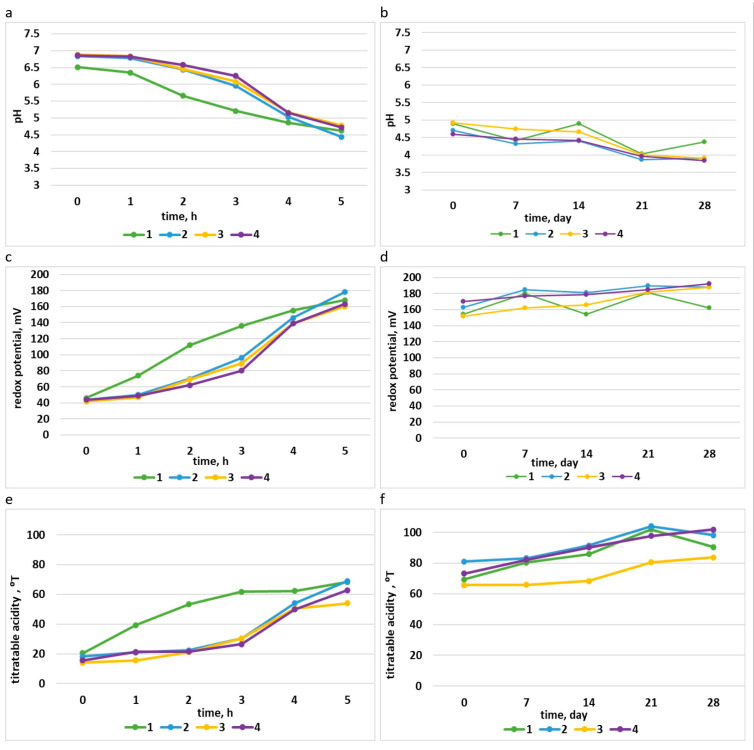
Parameters of the yogurt samples during the process of fermentation: (**a**) pH; (**c**) redox potential (mV); (**e**) titratable acidity (°T), and during storage: (**b**) pH; (**d**) redox potential (mV); (**f**) titratable acidity (°T).

Food products must meet certain quality requirements according to the relevant norms. Some of the main indicators of the fermentation process and product quality are pH, RP, and TA [27,28,55,61]. Many studies are performed for the evaluation of these parameters and other parameters that are important indicators of product quality and consumer acceptance [26,27,34,35,38,62,63]. In our case, the quality control of yogurt was performed by analyzing the physicochemical characteristics during the entire process. Figure 2 presents the results of pH, RP, and TA during the fermentation process and during storage at 4 °C for four types of yogurts, described in Table 1. The analysis showed that the pH decreased and RP and TA increased during fermentation and storage. The growth of LAB in yogurt causes the accumulation of organic acids, mainly lactic acid. Due to the increase in the content of lactic acid, the food matrix becomes acidic and the pH gradually decreases, while the concentration of hydrogen ions (H+) increases [45]. A first stage (4 h) was observed during the fermentation process, where the curves of yogurt samples with the strains continued to differ from that of yogurt sample 1. The second stage was the last hour of the fermentation process, where the curves of all yogurt samples were similar. The curves of the yogurt samples were generally similar during storage. The pH at the end of the fermentation process in all samples of yogurt was similar to that in the control, in the range of 4.4–4.8, and during storage, in the range of 3.8–4.8. RP also indicated that there were no large variations in the samples, and they were similar to the control. At the end of the fermentation process, the RP was in the range of 160–180 (mV), and during storage, in the range of 150–190 (mV). TA at the end of the fermentation process was similar to that in the control, i.e., 50–70 (°T). During storage, samples 2 and 4 had TA values similar to that in the control, but sample 3 had a lower TA. Similar results for yogurt parameters during the fermentation process were found in the study [38], and such changes during storage were also noticed in the study [35].

### 3.4. Water-Holding Capacity and Syneresis

One of the main properties of yogurt production is the formation of gel. The decrease in pH and denatured whey proteins cause the accumulation of casein. The accumulation of casein captures more water molecules. For yogurt, it is important to study this parameter as it is related to the texture of the product and is essential for microbial growth [64,65,66]. The results of WHC are demonstrated in Table 3. The sample with the highest WHC in the casein micellar structure, similar to the control yogurt, was the 3rd yogurt sample. Sample 4 was in between samples 2 and 3. No statistically significant difference in WHC during storage was observed among the yogurt samples. 

The instability of water in foods is illustrated by the separation of water being lost from gels, especially during low-temperature storage [66]. Syneresis, the phenomenon of water loss, is undesirable in yogurt. The results of syneresis are presented in Table 3, and a decrease in syneresis was observed from the first day to the 28th day of product storage. The yogurt samples with the lowest amount of whey released, i.e., similar to the control yogurt, were samples 3 and 4. In the first 7 days of storage, sample 4 released the lowest amount of whey. After the 7th day, sample 3 released the lowest amount of whey. No statistically significant difference in syneresis during storage was observed among the yogurt samples. All samples of yogurt were similar in WHC and syneresis, showing that the inoculated strains did not affect these parameters. Other studies have shown that the percentage WHC in yogurt is approximately similar to the values in this study [38,67], and for syneresis, similar results were found in other studies [3,68]. 

### 3.5. Rheological Characteristics (Viscosity)

**Figure 3:**
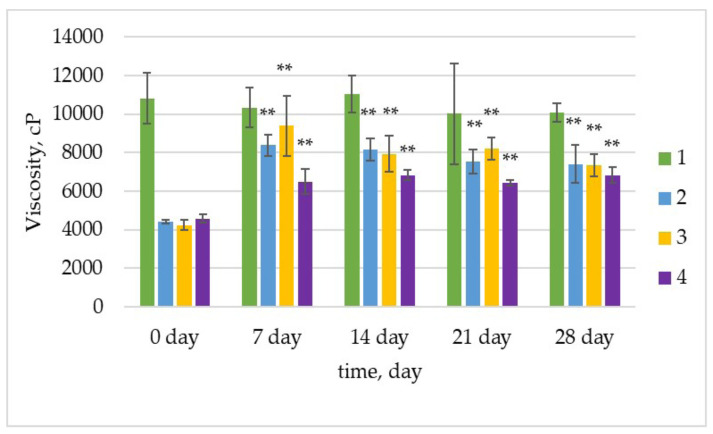
Apparent viscosity of yogurt samples during storage in cP. Error bars indicate the standard deviation of the means. One-way analysis of variance (ANOVA) was applied using the Tukey test for comparison of the means of yogurt samples during storage (** *p* < 0.01).

According to Mok et al., [64] as the protein gel is formed, the apparent viscosity increases rapidly and then reaches a plateau as the final network forms, entrapping the fat globules and residual serum. The increase in the structural strength of the protein network is considered to increase the apparent viscosity [64]. Viscosity results are presented in Figure 3. 

On day 0 of storage, the yogurt samples with LAB strains showed much lower viscosity than the control yogurt. The viscosity had a noticeable increase on the 7th day of storage compared to the 0th day of storage, approaching the value of the control, especially for sample 3 inoculated with strain *Lpb. plantarum*. Strains of *Lpb. plantarum* can produce exopolysaccharide (EPS) that has high thermal stability, which improves the structure of yogurt by increasing its viscosity [69]. In the study by Yang et al., Greek yogurt containing *Lpb. plantarum* had high viscosity [40]. Sample 4 inoculated with strains *L. delbrueckii* subsp. *bulgaricus* KZM 2-11-3 and *Lpb. plantarum* KC 5-12 had the lowest viscosity. In contrast, Yang et al. [40] reported that yogurt with *L. delbrueckii* subsp. *bulgaricus* had the lowest viscosity. These contrasting results can be interpreted by relying on interactions between binary cultures. No statistically significant difference was determined for yogurt sample 1 (*p* > 0.05). It can be emphasized that the viscosity that increased on the 7th day in samples 2, 3, and 4 was statistically different compared to the viscosity of the yogurt samples on day 0 of storage. Viscosity improved on the 7th day of storage for the three yogurt samples, maintaining the stability and approximately similar values until the 28th day. Similar results for the range of viscosity were found in other studies [35,39].

Factors that influence the fermentation process can affect the viscosity of the final product. As explained above, WHC is related to syneresis and viscosity and is important for product texture [30]. Further, the presence of different strains results in the production of acids at different rates and concentrations. The presence of acids affects the viscosity of the product, making viscosity a culture-dependent trait [70]. A Pearson correlation was carried out to determine the relationship between WHC, syneresis, pH, TA, and viscosity, and these variables had a very strong positive correlation, with a Pearson correlation coefficient (r) > 0.5.

### 3.6. Viability of Bacterial Strains in Yogurt

Probiotic products have the challenge of ensuring a sufficient number of viable cells until the time of consumption [71]. Table 4 shows the results of the total number of LAB during the storage time of yogurt. Viable cell counts of LAB were significantly increased for yogurt sample 1 within 7 days and 14 days of storage and in yogurt sample 2 within 7 days of storage. Viable cell counts of LAB in yogurt sample 2 and yogurt sample 3 decreased after 28 days of storage, with statistical significance compared with day 0 of storage. Despite this, the results showed the stability of the bacterial culture throughout the storage period and log10 (CFU/g) did not decrease to less than 8 until the end of the storage period. Therefore, the strains were alive and active and showed their functional and probiotic potential even on the 28th day. Similar results for the stability of bacteria in yogurt products were found in other studies [35,40,67,72,73]. Shori et al. [74] reported that all of the variants of fermented milk with Lactobacillus spp. showed the highest viable cell counts at 7 days of storage. There were no significant changes in the viable cell counts of Lactobacillus spp. during 21 days storage. Dimitrellou et al. [75] reported that probiotics in fermented milk grew well and retained their viability during four weeks storage. 

### 3.7. Sensory Characteristics

**Figure 4:**
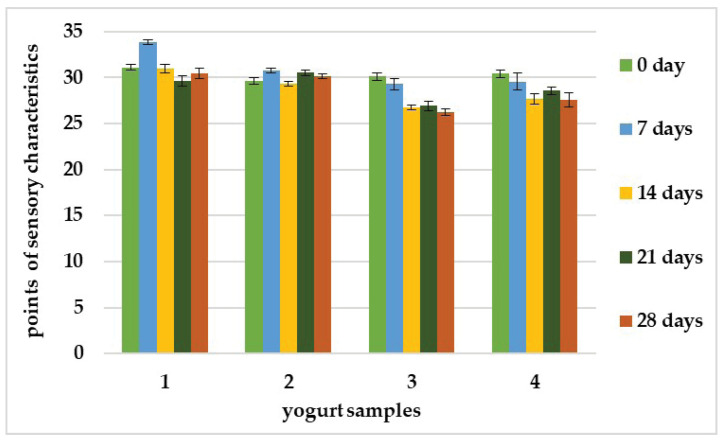
The average of the panelist’s total scores of the yogurt samples during storage. Error bars indicate the standard deviation of the means.

**Figure 5:**
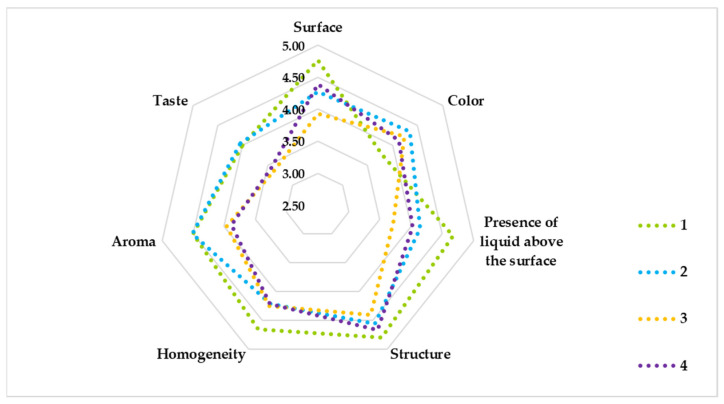
Evaluation of the sensory characteristics of the yogurt samples.

The assessment of sensory analysis serves to enable the distinction between types of products based on product characteristics such as surface, color, the presence of liquid above the surface, structure, homogeneity, aroma, and taste. For all samples, the characteristics of the product presented in the sensory analysis were evaluated, and the results are demonstrated in Figure 4 and Figure 5. The average total scores of the samples at different storage times are shown in Figure 4. All three samples had evaluation points very similar to that of sample 1 as a control variant. Samples 2 and 4 at four weeks of storage had more stable evaluation with very little deviation, while sample 3 has a decline after the 14th day.

The calculated averages of the product characteristics determined at five storage time points, i.e., 0 days, 7 days, 14 days, 21 days, and 28 days, are presented in Figure 5. Sample 2, with strain *L. delbrueckii* subsp. *bulgaricus* KZM 2-11-3 inoculated, had characteristics similar to those of the control yogurt. The panel evaluated this sample as having the best color, aroma, and taste. Sample 3, with strain *Lpb. plantarum* KC 5-12 inoculated, was evaluated for homogeneity. Sample 4, with both strains inoculated, generally had intermediate characteristics of those of samples 2 and 3. This sample had two characteristics similar to those in the control, the smooth and shiny surface and the structure. According to Coggins et al. [76], taste and texture are the factors that make the difference in the preference for yogurt. Aroma, sweetness, sourness, chalky mouthfeel, and viscosity were also identified as significant attributes in yogurt drinks [62].

## 4. Conclusions

Two strains of LAB, *L. delbrueckii* subsp. *bulgaricus* KZM 2-11-3, and *Lpb. plantarum* KC 5-12, were selected for this study. It is important to underline that the selection of the used strains was made based on the characteristics from previous studies showing that the two strains have probiotic potential and bioprotective properties. 

Four samples of yogurt were examined using two LAB strains, and the physicochemical, rheological, and sensory characteristics were studied. For all of the studied characteristics of the product, the samples of yogurt with the tested strains did not differ in most parameters from the control sample, especially at the end of the fermentation process and during the storage period. The strain *L. delbrueckii* subsp. *bulgaricus* KZM 2-11-3 was observed to have a strong effect in inhibiting the growth of *E. coli* in yogurt and also inhibited the growth of the yeasts *K. marxianus*, *K. lactis*, and *S. cerevisiae*, while the strain *Lpb. plantarum* KC 5-12 had a well-defined effect on yeast growth inhibition (greater than 60%). 

In conclusion, the use of *L. delbrueckii* subsp. *bulgaricus* KZM 2-11-3 and *Lpb. plantarum* KC 5-12 as strains with bioprotective and probiotic potential included in the composition of production starter cultures is very promising. With enriched starter cultures, healthy food products can be produced with preserved quality for the entire storage period. The presence of these strains as probiotics to enrich the starter culture in probiotic yogurt can bring benefits to human health with preserved quality of the product. 

## Figures and Tables

**Table 1:** Concentrations of selected strains and starter culture based on the milk volume.

		Strains (w, %)	
Yogurt Samples	Commercial Starter Culture (LB Bulgaricum) (w, %)	*L. delbrueckii* subsp. *bulgaricus* KZM 2-11-3	*L. plantarum* KC 5-12
1	1	-	-
2	0.1	5	-
3	0.1	-	5
4	0.1	2.5	2.5

**Table 2:** Percentage of yeast growth inhibition in the cell-free supernatant of two selected strains.

	Effect of Growth Inhibition of Yeasts, %		
Yeasts	Time for Inhibitory Effect, h	*Lactobacillus delbrueckii* subsp. *bulgaricus* KZM 2-11-3	*Lactiplantibacillus plantarum* KC 5-12
*Kluyveromyces* *marxianus*	72	84.5 ± 5.8	81.6 ± 5.8
*Kluyveromyces* *lactis*	48	69.3 ± 1.6	62.4 ± 1.4
*Saccharomyces* *cerevisiae*	72	31.6 ± 2.6	68.9 ± 8.0

The percentage yeast growth inhibition was calculated from yeast growth in the control. Data are expressed as the mean ± standard deviation.

**Table 3:** Percentages water-holding capacity and syneresis of the yogurt samples during storage.

WHC, %					Syneresis, %			
	Yogurt Samples				Yogurt Samples			
Storage Time	1	2	3	4	1	2	3	4
**0 day**	36.7 ± 0.02	35.1 ± 2.60	37.7 ± 3.37	35.5 ± 0.20	8.6 ± 1.50	12.6 ± 0.50	10.0 ± 3.25	9.4 ± 0.80
**7 days**	35.8 ± 0.96	33.3 ± 0.13	35.3 ± 0.74	34.3 ± 0.12	7.6 ± 0.80	12.9 ± 2.70	10.2 ± 0.70	7.8 ± 0.68
**14 days**	38.2 ± 0.92	36.5 ± 1.26	36.7 ± 0.96	35.8 ± 1.15	7.3 ± 0.95	10.0 ± 0.30	8.4 ± 0.00	9.6 ± 1.44
**21 days**	36.4 ± 0.89	34.3 ± 0.24	34.9 ± 0.94	34.3 ± 0.52	8.6 ± 1.80	8.8 ± 1.10	8.3 ± 1.20	10.3 ± 0.78
**28 days**	36.7 ± 2.44	35.1 ± 2.17	37.7 ± 2.13	35.5 ± 2.11	6.7 ± 1.05	9.9 ± 0.85	6.7 ± 0.00	6.9 ± 3.44

The percentage water-holding capacity and syneresis are expressed as the mean ± SD of triplicate samples. One-way analysis of variance (ANOVA) was applied using the Tukey test for comparison of the means of yogurt samples during storage (*p* > 0.05).

**Table 4:** Viability of total LAB in MRS agar during the storage of yogurt samples at 4 °C for 28 days.

Yogurt Samples	Log10 (CFU/g)				
	0 Day	7 Days	14 Days	21 Days	28 Days
1	8.55 ± 0.05	8.75 ± 0.02 **	8.77 ± 0.04 **	8.59 ± 0.07	8.47 ± 0.10
2	8.62 ± 0.09	8.78 ± 0.07 *	8.69 ± 0.05	8.52 ± 0.04	8.41 ± 0.06 *
3	8.81 ± 0.12	8.84 ± 0.06	8.79 ± 0.05	8.71 ± 0.04	8.50 ± 0.04 *
4	8.54 ± 0.05	8.58 ± 0.22	8.61 ± 0.10	8.58 ± 0.03	8.20 ± 0.08

The values of log10 (CFU/g) are the means of triplicate measurements ± standard deviation. One-way analysis of variance (ANOVA) was applied using the Tukey test for comparison of the means of yogurt samples during storage (** *p* < 0.01 and * *p* < 0.05).

## Data Availability

Data is contained within the article.
